# Advances in Spatial Transcriptomics for Infectious Disease Research: Insight for Vaccine Development

**DOI:** 10.3390/vaccines14020158

**Published:** 2026-02-07

**Authors:** Taehwan Oh

**Affiliations:** Department of Microbiology, College of Medicine, Dankook University, Cheonan 31116, Republic of Korea; ohth@dankook.ac.kr; Tel.: +82-041-550-3994

**Keywords:** spatial transcriptomics, virus, bacteria, parasites, vaccine development

## Abstract

Spatial transcriptomics (ST) enables genome-wide gene expression profiling while preserving tissue architecture, bridging the gap between bulk, single-cell, and histological analyses. Originating in 2016 and rapidly evolving since, ST has transformed infectious disease research by mapping host–pathogen interactions directly within intact tissues. Current platforms fall into two categories: sequencing-based methods (Visium, GeoMx, Stereo-seq) offering whole-transcriptome coverage at modest resolution and imaging-based platforms (Xenium, CosMx, MERFISH) providing single-cell or subcellular detail with targeted gene panels. These technologies reveal spatially organized immune responses, local tissue remodeling, and pathogen niches across viruses, bacteria, and parasites. In viral infection, ST uncovered heterogeneity in COVID-19 lung microenvironments, spatial immune activation in lymphoid tissues, and variant-specific inflammatory patterns. In bacterial disease, ST delineated granuloma architecture in tuberculosis and mapped vaccine-induced lung responses in Shigella studies. Parasitic infection studies identified localized inflammatory hotspots and microenvironmental control of T-cell differentiation in malaria. Despite powerful insights, ST faces constraints including RNA quality limitations, tradeoffs between resolution and transcript breadth, high cost, and analytical complexity. Nonetheless, ST increasingly informs vaccine design by identifying tissue-specific immune programs and protective microenvironments and is poised to become a standard tool for infectious disease biology.

## 1. Introduction

Spatial transcriptomics (ST) is a suite of technologies that enables genome-wide gene expression profiling while retaining the spatial location of cells within intact tissue sections [1]. Unlike bulk RNA sequencing, which averages signals across mixed cell populations, or single-cell RNA-seq, which dissociates cells and loses positional information, ST maintains tissue context [2]. The first demonstration of ST was in 2016 by Lundeberg and colleagues, and in 2021, Nature Methods recognized spatial methods as “Method of the Year” [3]. The original 2016 ST method by Lundeberg et al. consisted of reverse-transcription probes printed on slides, allowing tissue-wide RNA-seq with positional barcodes. These approaches have since been applied broadly (cancer, development, and pathology). In the context of infectious diseases, ST can map host–pathogen interactions in situ—for example, showing how immune cells, infected cells, and stromal cells are organized in lesions or infected organs [4]. This capability offers new insights into disease pathology and immune responses that cannot be obtained from bulk or single-cell studies alone.

Prior to spatial transcriptomics, infectious disease gene expression studies relied on bulk or dissociated approaches. Microarrays and bulk RNA-seq were widely used in the early 2000s to compare infected vs. uninfected tissues; these studies identified many differentially expressed genes but could not resolve which cells in the tissue produced them [5]. Researchers then turned to single-cell RNA-seq (scRNA-seq), which revolutionized immunology by cataloging cell types and states in infection [6,7]. However, scRNA-seq requires tissue dissociation, so information about where each cell was located in the organ is lost. Some intermediate methods partially addressed this gap. Laser capture microdissection (LCM) allowed isolation of specific regions for targeted sequencing, but it was laborious and had low throughput. RNAscope and HCR-FISH provided spatial information for a handful of genes at single-molecule resolution, but each experiment was limited to a few transcripts. In contrast, spatial transcriptomics extends these approaches by simultaneously profiling thousands of genes in situ. Conceptually, ST bridges the gap between scRNA-seq and histology, as it overcomes the loss of the topological information caused by tissue homogenization and cell dissociation in bulk or single-cell sequencing [5].

**Figure 1:**
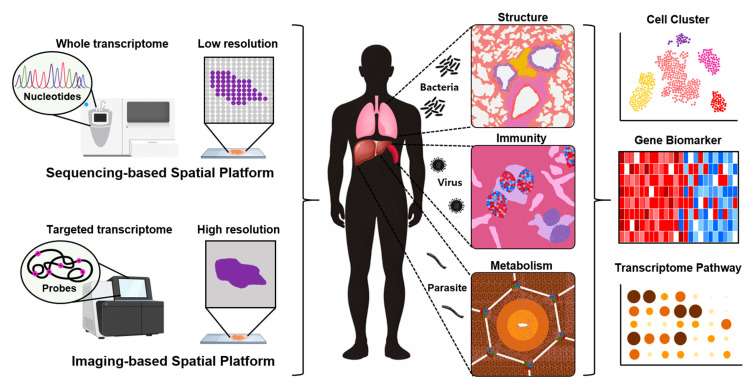
Workflow of spatial transcriptomics in infectious disease research. The colors in the cell cluster plot represent cell types. The colors in the gene biomarker heatmap represent the expression levels of each gene. The circle sizes and colors in the transcriptome pathway graph represent significance levels and enrichment scores of the gene set.

Indeed, ST can be seen as the latest step in a technological evolution: from bulk to single-cell to spatial, each advance adding more detail to our picture of pandemic and endemic diseases biology while revealing new types of information about where and how immune responses occur. For example, in acute infections like COVID-19, spatial profiling of the lung has revealed stage-dependent changes in immune cell composition and revealed “hotspots” of viral RNA and immune activation [8,9]. Similarly, spatial profiling of chronic infections such as tuberculosis (TB) can delineate the cellular architecture of granulomas [10]. This review offers opportunity for understanding ST layout, which allows researchers to correlate transcriptional programs with local histology and cell–cell interactions, making it a powerful tool for investigating infectious pathogenesis and for identifying tissue-specific immune responses that are relevant to vaccine design. The workflow of spatial transcriptomics in infectious disease research begins with selecting a platform appropriate for the experimental purpose, followed by identification of the pathogen’s target tissue and bioinformatic downstream analysis (Figure 1).

## 2. Current Platforms and Features

Spatial transcriptomics platforms can be broadly categorized according to their underlying detection methodology, next-generation-sequencing (NGS)- or fluorescence-in situ-hybridization (FISH)-based, and each approach requires distinct computational pipelines [1,2,3]. NGS-based commercial platforms, such as 10X Visium, BGI Stereo-seq or Nanostring GeoMX generate spatially indexed cDNA libraries from discrete spot arrays or regions of interest (ROIs). These libraries were subjected to whole-transcriptome sequencing, followed by normalization and dimensionality reduction principal component analysis (PCA). Differential gene expression (DGE) and gene set enrichment analysis (GSEA) are subsequently performed to identify spatially enriched molecular programs and transcription factors. In contrast, FISH-based platforms, such as 10X Xenium, Nanostring CosMx, and Vizgen MERSCOPE, use in situ probes to detect specific transcripts in single-cell or subcellular compartments. Following fluorescence signal acquisition, gene expression data are normalized and clustered using techniques such as uniform manifold approximation and projection (UMAP). Additional downstream analyses include trajectory inference and cell–cell interaction mapping, which reveal localized functional states and lineage relationships [11,12]. Currently available commercial platforms of spatial transcriptomics were summarized below (Table 1).

• BGI Stereo-seq (Shenzhen, China)—A DNA-nanoball (DNB)-based spatial transcriptomics platform that achieves ultra-high spatial resolution using patterned arrays. Tissue sections are placed onto chips densely coated with millions of DNA nanoballs carrying unique spatial barcodes. mRNAs hybridize in situ and are reverse-transcribed, and cDNA molecules are amplified and sequenced. Because DNB barcodes are spaced at submicron scale (≈500 nm), Stereo-seq provides true near-single-cell to subcellular resolution over very large tissue areas (centimeter-scale), enabling whole-organ or whole-embryo maps. The method is high-sensitivity and supports genome-wide profiling, but requires highly specialized chips and BGI’s proprietary sequencing workflow, limiting accessibility compared with more widely adopted platforms.
• 10x Genomics Visium (Pleasanton, CA, USA)—A sequencing-based “slide” method. Fresh-frozen and formalin-fixed, paraffin-embedded (FFPE) tissue sections are placed on glass slides printed with an array of spots bearing barcoded oligo (dT) capture probes. mRNAs bind in situ and are reverse-transcribed with spatial barcodes, then pooled and sequenced. The commercial Visium platform (launched in 2018) uses 55 μm spots (improved from the original 100 μm) and can assay ~5000 transcripts per spot. A new Visium HD array (2024) further reduces spot size to ~2 μm, enabling nearly gapless, near-single-cell coverage. Visium is high-throughput and genome-wide, but each spot contains multiple cells (limiting exact single-cell resolution) and requires specialized equipment.
• NanoString GeoMx (Seattle, WA, USA)—Not a whole-transcriptome method, but a targeted approach using oligo-tagged probes and UV photocleavable barcodes. It profiles more than thousands of transcripts in regions of interest (user-defined ROIs) on either frozen or FFPE sections. GeoMx allows flexible region selection and has been used to study infection in archived clinical samples but requires specialized equipment and is limited to pre-defined targets.
• Vizgen/MERSCOPE (Cambridge, MA, USA)—An imaging-based approach using combinatorial fluorescent probes. First reported in 2015, MERFISH labels hundreds to thousands of transcripts in situ by sequential hybridization with error-correcting barcodes. It achieves single-molecule sensitivity and cellular resolution, enabling thousands of genes to be profiled in the same section. Its tradeoff is that it requires sophisticated microscopy and image processing and typically targets a pre-selected panel of genes.
• 10x Genomics Xenium (Pleasanton, CA, USA)—A fluorescence-based in situ transcriptional profiling platform that detects RNA molecules directly inside intact tissue sections using targeted probe panels. Xenium employs rolling-circle amplification to generate fluorescent “amplicon dots” at each transcript’s location, followed by iterative imaging cycles to decode gene identities. Current panels cover hundreds to thousands of genes, with subcellular (~200–300 nm) localization precision and single-cell resolution across relatively large tissue areas. Xenium preserves tissue morphology for multimodal analysis (e.g., H&E, IF co-staining). Because it is targeted rather than whole-transcriptome, gene coverage is limited by probe design, and the instrument requires a dedicated imaging and chemistry system.
• NanoString CosMx SMI (Spatial Molecular Imager, Seattle, WA, USA)—A multiplexed in situ RNA imaging platform that uses oligo-labeled probes and cyclic fluorescent readout to detect transcripts directly within preserved tissue at single-molecule resolution. CosMx decodes targets via sequential hybridization and imaging cycles, enabling detection of up to ~6000 RNA targets (and 100+ proteins) with subcellular precision, capturing transcript locations within cell bodies and processes. It delivers true single-cell and subcellular maps across moderately large fields of view and supports FFPE as well as fresh-frozen samples. As a targeted method, its gene panel breadth is limited by probe design, and the workflow requires specialized instrumentation and long imaging cycles, but it provides very high spatial resolution and rich multimodal data.

Each platform presents unique tradeoffs among resolution, transcriptomic breadth, and spatial scale. NGS-based methods provide unbiased whole-transcriptome coverage but are limited by relatively coarse resolution (1–10 cells per spot). In contrast, FISH-based techniques offer single-cell or subcellular resolutions, albeit with a restricted number of detectable transcripts per assay. Furthermore, differences in analytical scan size across platforms necessitate careful consideration of tissue dimensions and species-specific anatomy when selecting spatial transcriptomic strategies. Generally, ST methods allow correlation of gene expression with histological features in various organ, but achieving both high spatial resolution and full-transcriptome depth remains challenging.

## 3. Applications on Pathogen Types

Spatial transcriptomics has transformed the study of infectious diseases by mapping gene expression within intact tissues, revealing how viruses, bacteria, and parasites reshape local microenvironments. Across pathogens—from SARS-CoV-2 to *Mycobacterium tuberculosis* and *Plasmodium*—ST has uncovered hidden immune niches, tissue pathology, and spatially confined host–pathogen interactions that were previously inaccessible. Collectively, the findings demonstrate that ST not only advances mechanistic understanding of in vivo infection biology but also provides critical insights for vaccine design and targeted therapeutic strategies (Table 2).

### 3.1. Virus

Spatial transcriptomics has been used to dissect viral infections in tissue, particularly respiratory viruses. During the COVID-19 pandemic, several studies applied ST to post-mortem or animal lung tissues. For example, a spatial atlas of SARS-CoV-2–infected lung showed extensive remodeling of epithelial, immune, and stromal compartments, revealing disease stages linked to severity, including defective pneumocyte differentiation and fibroblast expansion [13]. Desai et al. observed that viral RNA and host responses were highly heterogeneous in space and time; some regions of lung showed high viral load and interferon responses while others did not [9]. Recent advances in the ST platform allowed the investigation of an animal model system. Dinnon et al. used spatial profiling (GeoMx DSP and RNA FISH) in a mouse model to compare acute versus long COVID stages, identifying distinct gene signatures at each stage [14]. Other studies (Oh et al.) performed spatial profiling on autopsy lungs from macaques experimentally infected with diverse clade of SARS-CoV-2, discovering that immune signaling were heterogeneously distributed among variants infection models [15,16]. Their works defined transcriptional alterations in specific pulmonary microstructures (alveoli and blood vessel), with limited number of differentially expressed genes involved in cytokine and cell damage responses in bronchiolar region. Further, spatially resolved transcriptome were used to effectively demonstrate systemic and local immune response, by illustrating the spatiotemporal cellular dynamics of the germinal center of mediastinal lymph nodes and bronchus-associated lymphoid tissue (BALT) of the lung during the recovery process after the viral infection [17,18].

Outside COVID-19, ST has also been explored in other viral contexts. Spatial analysis of mouse lung following influenza infection show pathological features related to stem-cell loss in alveolar epithelium and hyperplastic basal cell differentiation resulting loss of lung function [19]. Other studies compared lung injury from H1N1 with SARS-CoV-2 and identified unique transcriptional signatures for each pathogen’s acute respiratory distress syndrome (ARDS) phase in alveolar epithelium, vascular tissue, and macrophage regions [20]. Integrated with bulk and singe cell sequencing analyses, spatial transcriptomics were also used to identify profibrotic and antifibrotic biomarkers expressed by pulmonary epithelium, showing the complex microenvironments that enhance fibrosis in aged-induced changes [21].

High-resolution spatial transcriptomics revealed that transcriptionally active hepatitis B Virus (HBV) integration into the chromosomes of infected hepatocytes is widely dispersed across the liver, and map to oncogenesis-related host genes. This spatially resolved analysis also showed that antiviral therapy markedly reduced the density and distribution of these integration events, highlighting its potential to limit HBV-driven genomic alterations [22]. Using sequencing-based GeoMx platform (Seattle, WA, USA), the other study mapped region-specific gene expression in liver biopsies from HBV patients with human immunodeficiency virus (HIV) coinfection, highlighting disrupted hepatocyte function and distinct immune signatures linked to coinfection type [23].

ST is also widely used to uncover the complex immune landscape of the affected organ during chronic HIV infection. ST delineate the immune regulatory microenvironment of HIV-infected lymph node follicles, a key reservoir site. ST enabled precise mapping of cellular interactions and regulatory pathways—specifically implicating the HLA-E–NKG2A axis in limiting antiviral effector functions. These findings underscore ST a powerful tool for uncovering spatially confined immune evasion mechanisms driving HIV persistence within tissue reservoirs [24]. In ectocervix, by spatially mapping gene expression within epithelial and submucosal compartments, the ST approach revealed tissue-wide immune activation, including distinct B-cell- and T-cell-mediated responses, a persistent antiviral interferon signature, and structural epithelial alterations [25].

Altogether, these spatial studies have pinpointed pathological features (microvascular damage and fibrotic changes) and local immune niches (germinal center and BALT structure) and that drive disease, highlighting how ST can uncover in vivo virus–host dynamics. These spatial atlases have directly informed potential personalized treatment therapies targeting specific immune pathways and provided context for vaccine effects on systemic pathology.

### 3.2. Bacteria

Bacterial infections that create complex tissue lesions are research targets for ST. A prime example is tuberculosis, where *Mycobacterium tuberculosis* induces granulomas, spherical aggregates of infected macrophages and other immune cells. The spatial organization of tuberculosis (TB) granulomas critically influences infection outcome, but the underlying cellular programs were poorly understood. Recent ST efforts have begun to fill this gap [10]. For instance, a spatial multi-omics study (combining antibody imaging, ST, and scRNA-seq) in macaque and human granulomas delineated immunometabolic zones within each lesion—a hypoxic, necrotic core enriched in immunosuppressive macrophages, surrounded by rings of IFN-γ^+^ T cells [26]. In parallel, paired single-cell and spatial RNA-seq of human and zebrafish TB granulomas identified a dominant macrophage subset (marked by osteopontin/SPP1) that forms a concentric layer around necrosis, suggesting these macrophages are central regulators of granuloma structure [27]. ST was also used to unravel the complex immune and structural dynamics in the lung during early HIV and *Mycobacterium tuberculosis* (Mtb) coinfection. By spatially mapping gene expression, the analysis revealed infection-specific immune infiltration patterns, including high CD4^+^ T-cell accumulation in Mtb infection but not in HIV coinfection. ST further identified lymphoid cell aggregates in coinfected lungs with unique transcriptional signatures resembling granulomas, uncovering early morphological and immunological remodeling not apparent through previous conventional methods [28].

Recent study used sequencing-based Visium ST to examine immune responses in mice vaccinated with an experimental intranasal Shigella vaccine (L-DBF fusion protein from *Shigella flexneri*) [29]. Spatial maps showed that vaccinated lungs had higher expression of B-cell and T-cell markers, and unexpected roles for non-immune cells: fibroblasts shifted toward immune-modulatory gene expression and cardiomyocytes upregulated leukocyte-recruiting signals. This revealed that vaccination not only boosts classical immunity but also reprograms structural lung cells.

These spatial transcriptomics studies revealed how immune and stromal cells are arranged in the lesions, and identified molecular pathways that may be exploited by the bacteria. These insights also have direct implications for vaccine research, understanding which cell types and zones contain the bacteria or primed immune cells can guide antigen delivery strategies or host-targeted therapies.

### 3.3. Parasites

Parasitic infections often involve specialized tissue stages where localized host–pathogen interactions occur. The liver stage of malaria is a key example, as it is targeted by leading vaccine candidates. A recent study used ST on *Plasmodium berghei*–infected mouse livers across multiple time points [30]. The authors generated a high-resolution spatiotemporal atlas of malaria liver infection, showing inflammatory hotspots—distinct tissue regions with high immune cell infiltration and upregulated inflammatory genes—that appeared near parasite-infected hepatocytes. These hotspots were enriched for phagocytic and interferon pathways, suggesting focal points of host defense. The study also detected upregulation of lipid metabolism genes at infection sites, showing that the parasite may alter local metabolism. Also, this study suggests that ST can capture parasite RNA within host tissue, highlighting how host and parasite interactions manifest in situ. Another recent study examined plasmodium infection in the spleen by tracking CD4^+^ T-cell differentiation [31]. Using a high-plex spatial transcriptomics approach at near single-cell resolution, it showed that T helper 1 (Th1) cells and T follicular helper (Tfh) cells occupied distinct splenic microenvironments. Specifically, Th1 cells were found mainly in the red pulp co-localized with activated monocytes, whereas Tfh-like cells clustered in B-cell follicles with stromal cells. This spatial segregation suggests that microstructural context influences T-cell fate and function during malaria, and reveals signaling that reinforce Th1 effector differentiation.

These examples show that ST can map both tissue-resident and migratory immune responses in parasitic infections. Such maps are invaluable for vaccine strategies: understanding the liver’s localized immune response with cellular dynamics could inform strategies to boost liver-stage immunity. Also, how malaria primes different T-cell fates could inform vaccines that seek to elicit particular T helper responses. Therefore, spatial insights might identify which cell types or locations to target with vaccines or immunotherapies.

## 4. Limitations and Challenges

ST offers unprecedented spatial insight, but faces several technical and analytical challenges. Proper experimental design and complementary methods are often needed to corroborate spatial findings.

First of all, ST requires high-quality RNA in tissue sections. Some tissues are difficult to preserve; for example, lungs with large air spaces must be frozen rapidly to avoid RNA degradation, and heavily pigmented or fibrotic areas can interfere with probe binding [32,33]. FFPE tissues—a staple of pathology—are now partially accommodated (Visium FFPE and NanoString DSP protocols exist), but RNA from FFPE is often degraded and variable. This issue is particularly highlighted when setting fixation conditions of the tissues infected with pathogens that require high biosafety levels. In particular, most NGS-based platforms recommend about 1–2 days of formalin fixation and 1 day of overnight tissue processing and immediate embedding to preserve RNA quality. Thus, sample handling protocols must be carefully optimized for each infection model and applied ST platform coverages.

Spatial resolution and sensitivity are also other issues when we design ST analysis. Most sequencing-based ST methods capture multiple cells per spot. For instance, the standard Visium spot (~55 μm) typically covers several cells, blurring single-cell level. Newer high-definition arrays (Visium HD and Stereo-seq) push toward subcellular resolution, but at present there are still gaps between spots or limits in coverage. Similarly, imaging methods (MERFISH, seqFISH) can reach single-cell resolution, but they generally profile fewer genes and require complex instrumentation and probe synthesis. In practice, no method yet achieves both true single-cell resolution and unbiased whole-transcriptome coverage. This limitation means that detected transcripts are often averaged over small cell clusters, which complicates interpretation and may miss rare cell types in the site of infection. Currently, a complementary method is to derive key marker genes from whole-transcriptome data obtained through NGS, then design in situ probes for those genes to delineate target cells in imaging-based platforms.

Many ST protocols capture only the 3′ end of polyadenylated transcripts and sequence short fragments. As a result, splice variants and non-polyadenylated RNAs (many bacterial transcripts) are underrepresented. Because most ST technologies recover only single-end transcript fragments rather than complete transcripts, they restrict detailed investigation of immune cell receptor repertoires and alternative splicing [34]. Moreover, lower-abundance transcripts including viral RNA are harder to detect spatially due to the limited sensitivity of capture. Complementary methods such as RNA in situ hybridization are required to develop the study.

Spatial data are high-dimensional and require specialized analysis tools. Integrating ST with scRNA-seq (for cell-type deconvolution) or with histology images (for annotation) adds layers of complexity. Algorithms for identifying spatial domains or cell–cell interactions are still being developed. There is a risk of overinterpreting spatial patterns without adequate controls, and distinguishing true biological variation from technical noise can be challenging. Standardized pipelines for ST analysis are maturing but remain less established than bulk or scRNA-seq workflows. Integration and validation with the previous workflows are still needed. For example, even in the first step of analysis, assigning cell-type identity to spatial spots often relies on correlation with known marker genes or external scRNA-seq data.

Commercial ST platforms are expensive and require dedicated instruments and reagents. Imaging-based ST requires advanced microscopes and extensive imaging time. As a result, large cohorts including many patient samples or replicates in animal model samples can be impractical to analyze spatially. It is necessary to introduce a tissue microarray technology that arranges key lesion cores from multiple tissue blocks into a single recipient block and allows simultaneous ST analysis of multiple cores on the single slide. High computational resources are also needed for the large datasets. These factors slow the adoption of ST compared with other transcriptomic technologies.

## 5. Conclusions

Spatial transcriptomics is rapidly transforming infectious disease research by illuminating the landscape of host–pathogen interactions in tissue. By providing maps of where specific immune cells, cytokines, antibodies and pathogens are located, ST has revealed novel applications of molecular biology for infectious diseases. These spatial insights have direct implications for vaccines. Knowing which cell types drive protective immunity at the site of infection can guide antigen selection and delivery methods. ST studies in COVID-19 delineated germinal center B-cell dynamics with gene signatures in virus-infected microstructures, suggesting potential immune changes in application of vaccine candidates [17,18]. Also, the Shigella vaccine study explicitly showed that spatially mapped immune activation including B-cell and T-cell markers in lung could be linked to protective outcomes, highlighting ST data could inform vaccine optimization [29]. In the malaria liver, ST may highlight antigenic pathways upregulated during infection, pointing to potential vaccine targets in hepatocytes or Kupffer cells [30]. These studies demonstrated a novel approach to analyzing resident immunity formed at the site of infection, in line with the recent trend of vaccine development to focus on local defense of the target system rather than on sensitizing systemic immune [35].

There are still key questions that have not been addressed in infectious disease research. Investigating the heterogeneous distribution of pathogens in affected tissues, comparing transcriptional phenotypes of cells in virus-infected microstructures versus intact microstructures, as well as examining the surrounding cellular neighborhoods, may reveal the underlying factors driving this selective evasion mechanism. This approach could serve as a driving force for the strategy of vaccine development. To assess how novel vaccines influence the infection and host responses, ST can be applied to explants treated with or without the new vaccine candidates. This enables the characterization of changes in cell types, quantities, and distributions, as well as alterations in ligand–receptor interactions or intercellular networks. Conclusively, ST research in the field of infectious diseases can contribute to increasing vaccine targeting while reducing side effects.

Looking ahead, we expect continued advances: higher-throughput and higher-resolution platforms will become more common. Integration of spatial transcriptomics with protein imaging (spatial proteomics) and other omics (spatial epigenomics) will provide multidimensional tissue atlases [36,37,38,39]. Importantly, as costs fall and methods develop, spatial profiling may move into clinical research—for example, diagnosing infection states in patient biopsies or evaluating vaccine responses in tissue samples.

In summary, spatial transcriptomics has filled a critical gap left by earlier methods. It enables us to see where genes are expressed within infected tissues, not only if they are expressed. This spatial dimension is unlocking a deeper understanding of pathogenesis and immunity. With further technological and analytical improvements, ST is poised to become a standard tool in vaccine development and infectious disease biology, yielding richer and more actionable insights into host–pathogen interactions.

## Figures and Tables

**Table 1:** Commercial spatial transcriptomics platforms and features.

	Company/Platform	Transcript Coverage	Resolution	Scan Area (mm^2^)
NGS-based (In situ capture)	BGI/Stereo-seq	Whole transcripts	500 nm spot	10 × 10
	10X/Visium	Whole transcripts	2000 nm spot	6.5 × 6.5
	Nanostring/GeoMx	~18,000 transcripts	User-defined ROI	35.3 × 14.1
FISH-based (Imaging)	Vizgen/MERSCOPE	~1000 transcripts	100 nm (subcellular)	18 × 22
	10X/Xenium	~5000 transcripts	50 nm (subcellular)	12 × 24
	Nanostring/CosMx	~6000 transcripts	50 nm (subcellular)	20 × 15

**Table 2:** Spatial transcriptomics studies in the field of infectious diseases.

	Disease	Tissue	Application Context	Platform
Virus	COVID-19	Lung Lymph nodes	Inflammatory microenvironment Germinal center reaction	GeoMx Xenium
	Influenza	Lung	Stromal cell dynamics	Visium
	Hepatitis	Liver	Host genome and function alteration	Visium, GeoMx
	AIDS	Lymph nodes Ectocervix	Reservoir tissue immune evasion Persistent immune activation	Visium Visium
Bacteria	Tuberculosis	Lung	Granuloma structure organization	Visium
	Shigellosis	Lung	Vaccine-induced local response	Visium
Parasite	Malaria	Liver	Cell metabolism and differentiation	Visium, Stereo-seq

## Data Availability

No new data were created or analyzed in this study. Data sharing is not applicable to this article.

## Abbreviations

The following abbreviations are used in this manuscript:

ST	Spatial transcriptomics
scRNA-seq	Single-cell RNA sequencing
LCM	Laser capture microdissection
FFPE	Formalin-fixed paraffin-embedded
FISH	Fluorescence in situ hybridization
NGS	Next-generation sequencing
DGE	Differential gene expression
GSEA	Gene set enrichment analysis
UMAP	uniform manifold approximation and projection
ROI	Region(s) of interest
DNB	DNA nanoball
DSP	Digital spatial profiling
IF	Immunofluorescence
H&E	Hematoxylin and eosin
COVID	Coronavirus disease 2019
HBV	Hepatitis B virus
HIV	Human immunodeficiency virus
AIDS	Acquired immunodeficiency syndrome
ARDS	Acute respiratory distress syndrome
TB	Tuberculosis
Mtb	*Mycobacterium tuberculosis*
SARS-CoV-2	Severe Acute respiratory syndrome coronavirus 2
BALT	Bronchus-associated lymphoid tissue
Th1	T helper 1 cells
Tfh	T follicular helper cells
